# Elucidating the mechanistic relationships between peroxisome proliferator–activated receptors and hepatic fibrosis using the ROBOKOP knowledge graph

**DOI:** 10.3389/ftox.2025.1549268

**Published:** 2025-04-22

**Authors:** Karamarie Fecho, Nyssa Tucker, Jon-Michael Beasley, Scott S. Auerbach, Chris Bizon, Alexander Tropsha

**Affiliations:** ^1^ Renaissance Computing Institute, University of North Carolina at Chapel Hill, Chapel Hill, NC, United States; ^2^ Copperline Professional Solutions, LLC, Pittsboro, NC, United States; ^3^ UNC Eshelman School of Pharmacy and Curriculum in Toxicology and Environmental Medicine, University of North Carolina at Chapel Hill, Chapel Hill, NC, United States; ^4^ UNC Eshelman School of Pharmacy, University of North Carolina at Chapel Hill, Chapel Hill, NC, United States; ^5^ Division of Translational Toxicology, National Institute of Environmental Health Sciences, Durham, NC, United States; ^6^ Predictive, LLC, Raleigh, NC, United States

**Keywords:** knowledge graph, knowledge sources, semantic harmonization, reasoning algorithm, adverse outcome pathway, mechanistic toxicology, chemical safety

## Abstract

We developed the Reasoning Over Biomedical Objects linked in Knowledge Oriented Pathways (ROBOKOP) application as an open-source knowledge graph system to support evidence-based biomedical discovery and hypothesis generation. This study aimed to apply ROBOKOP to suggest biological mechanisms that might explain the hypothesized relationship between exposure to the herbicide and lipid-lowering drug clofibrate, an activator of peroxisome proliferator-activated receptor-α (PPARA), and hepatic fibrosis. We queried ROBOKOP to first establish that it could demonstrate a relationship between clofibrate and PPARA as a validation test and second to identify intermediary genes and biological processes or activities that might relate the activation of PPARA by clofibrate to hepatic fibrosis. Queries of ROBOKOP returned several paths relating clofibrate, PPARA, and hepatic fibrosis. One path suggested the following: *clofibrate – affects / increases_ expression_ of / increases_ activity_ of / increases_ response_ to / decreases_ response_ to / is_ related_ to – PPARA – is_ actively_ involved_ in – cellular response to lipid – actively_ involves – CCL2 – is_ genetically_ associated_ with – hepatic fibrosis*. This result established a relationship between clofibrate and PPARA and further suggested that PPARA is actively involved in the cellular response to lipids, which actively involves the chemokine ligand CCL2, a gene genetically associated with hepatic fibrosis; thus, we can infer that PPARA, upon activation by clofibrate, plays a role in hepatic fibrosis. We conclude that ROBOKOP can be used to derive insights into biological mechanisms that might explain relationships between environmental exposures and liver toxicity.

## 1 Introduction

Knowledge graphs (KGs) provide a powerful framework for knowledge representation and serve as a valuable tool for exploring established and inferred relationships between entities of scientific interest (Singhal, 2012). KGs are being applied in numerous scientific fields and industries, including commercial search engines, healthcare, finance, and entertainment (Dilmegani, 2024; Sajid, 2022; Zou, 2020). In a KG, “triples” or “subject–predicate–object” relationships are used to express core knowledge assertions or statements. In a biomedical KG, example triples might be “*(albuterol)–(treats)–(asthma)*,” “*(amoxicillin/clavulanic acid)–(causes)–(drug-induced liver injury)*,” or “*(perfluorooctanoic acid)–(is associated with)–(cancer)*.” The subject and object of each triple are represented as nodes within a KG, with the predicate represented as an edge between the subject and object that describes the relationship between those entities. Additional information on the core assertion may be captured as node or edge properties, edge attributes, or statement qualifiers.

The Reasoning Over Biomedical Objects linked in Knowledge Oriented Pathways (ROBOKOP) application was developed by our team as an open-source, biomedical, KG-based system to support evidence-based biomedical discovery and hypothesis generation (Bizon et al., 2019; Morton et al., 2019). ROBOKOP has been applied to cases across numerous biomedical domains and application areas, including environmental health, drug discovery, and other application areas (Fecho et al., 2021; Korn et al., 2022). In this study, we describe ROBOKOP and its application to a case seeking to determine how environmental exposures affect liver toxicity. We focus specifically on the herbicide and lipid-lowering drug clofibrate (National Center for Biotechnology Information, 2024a), an activator of peroxisome proliferator-activated receptor-α (PPARA) (Decara et al., 2020), and hypothesize that the activation of PPARA by clofibrate is related to hepatic fibrosis. We based our hypothesis on a conceptual pathogenic model of hepatic fibrosis that was developed by Kim and Lee (2018). In brief, the authors proposed a “multiple-parallel hit” model that is framed as an adverse outcome pathway (AOP) (Ankley et al., 2010) and asserts that environmental exposures and/or metabolic factors increase free fatty acids (endogenous ligands of PPARA; Varga et al., 2011) and liver metabolites in hepatocytes, leading to hepatic injury, which then stimulates an inflammatory response in Kupffer cells and a fibrotic response in hepatic stellate cells, leading to hepatic fibrosis. We aimed to apply ROBOKOP to substantiate our hypothesized relationship between PPARA activation and hepatic fibrosis by replicating the basic features of the pathogenic model of hepatic fibrosis put forward by Kim and Lee (2018) and extending the model to the PPARA activator clofibrate.

## 2 Materials and methods

ROBOKOP is comprised of a user interface (UI), including a ROBOKOP question-builder tool; a ROBOKOP KG; a collection of harmonized and interoperable knowledge sources represented as KGs within a ROBOKOP service termed Automat; and a variety of tools for directly exploring and programmatically querying the ROBOKOP KG or its components. ROBOKOP ingests, integrates, and semantically harmonizes data from nearly 40 underlying knowledge sources. The application uses an upper-level data model termed the Biolink Model (Unni et al., 2022) for data representation and harmonization. ROBOKOP also standardizes synonymous node names and identifiers from separate data sources and ontologies using the Node Normalization service (NCATS Translator, 2024b), which exposes identifier cliques created by a tool called Babel (NCATS Translator, 2024a). The ROBOKOP KG currently contains ∼10 million nodes and ∼140 million edges.

Users can pose queries to ROBOKOP in multiple ways, including via the ROBOKOP question-builder web interface to the ROBOKOP KG. After a query is posed to the question-builder tool, the ROBOKOP reasoning algorithm (ROBOKOP Reasoning, 2025) searches the underlying KG to identify the answer subgraphs that match the general topology of the query. A ROBOKOP answer-ranking algorithm (Morton et al., 2019) is then applied to the complete result set. The algorithm applies weights to each edge within each answer subgraph based on edge metrics that describe the strength of the relationship (e.g., *p-*values) or the number of supporting publications. The supporting publications are provided by either the knowledge source(s) that provided the edge and/or by a service termed OmniCorp (ROBOKOP Reasoning, 2025), which provides the co-occurrence frequency of the edge subject and object nodes within PubMed abstracts. OmniCorp’s *biolink:occurs_together_in_literature_* with edges are added to the results to provide supporting evidence. For scoring, ROBOKOP’s answer-ranking algorithm weighs the publications derived from the curated knowledge sources more heavily than those derived from OmniCorp. The final answer subgraph is then treated as a resistance network and assigned a score that is inversely related to the resistance of the overall network.

We aimed to apply ROBOKOP to support and explain our hypothesized relationship between PPARA activation and hepatic fibrosis by replicating the basic features of the pathogenic model of hepatic fibrosis put forward by Kim and Lee (2018) and extending the model to the PPARA activator clofibrate. We used the ROBOKOP question-builder tool to explore connections between clofibrate, PPARA, and hepatic fibrosis. Specifically, we queried ROBOKOP to first establish that ROBOKOP could demonstrate a relationship between clofibrate and PPARA as a validation test and then to identify intermediary genes and biological processes or activities that might relate the activation of PPARA by clofibrate to hepatic fibrosis.

## 3 Results

**FIGURE 1:**
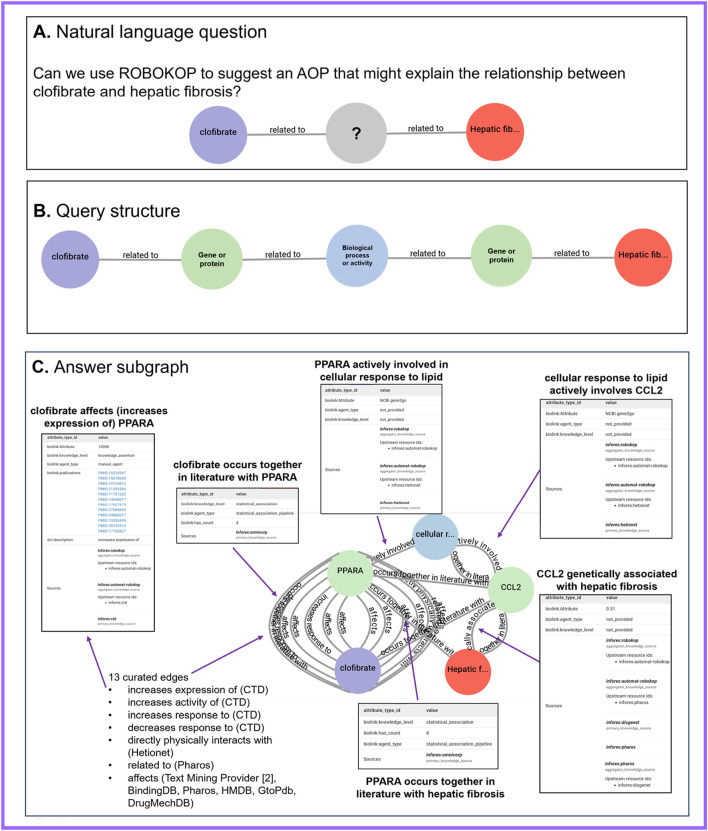
ROBOKOP results suggesting biological processes and activities that might explain the hypothesized relationship between clofibrate and hepatic fibrosis. **(A)** Natural language question, which asked whether we could use ROBOKOP to explain the relationship between clofibrate and hepatic fibrosis. **(B)** General query structure that we identified in response to the question in **(A)**. **(C)** Example answer subgraph that matched the query structure in **(B)** and contained 22 total edges, with 16 edges contributed by curated knowledge sources and six co-occurrence edges contributed by ROBOKOP’s OmniCorp service. Note that the *biolink:occurs_together_in_literature_with* edges were not part of the original query but were rather added to the results by OmniCorp to provide additional supporting evidence for use in ROBOKOP’s answer-ranking algorithm. Selected edges are highlighted, along with full confidence, evidence, and provenance, which were obtained by clicking on each edge. ROBOKOP, Reasoning Over Biomedical Objects linked in Knowledge Oriented Pathways.

We posed the following natural-language question: *“Can we explain the relationship between clofibrate exposure and hepatic fibrosis?”* (Figure 1A). To answer this general question using ROBOKOP, we ran a series of queries that focused on clofibrate and hepatic fibrosis and resulted in a query that was structured generally as follows: *clofibrate - related_to - Gene Or Protein - related_ to - Biological Process or Activity - related_ to - Gene Or Protein - related_ to - hepatic fibrosis* (Figure 1B). In this query, “clofibrate” is specified as a Biolink Model class *biolink:ChemicalEntity* and “hepatic fibrosis” is specified as a Biolink Model class *biolink:PhenotypicFeature*. The *biolink:BiologicalProcessOrActivity* and *biolink:GeneOrProtein* classes are not specified. The *biolink:related_to* predicate is the root of the Biolink Model predicate hierarchy, and so, queries using this predicate will return more specific predicates when available in the ROBOKOP KG. The general structure of the query was intended to resemble a basic AOP and maximize node connectedness within the ROBOKOP KG as the non-specified intermediary nodes tend to be highly represented within the graph.

ROBOKOP returned several answer subgraphs that matched this general structure. One answer subgraph described the following: *clofibrate – affects / increases_ expression_ of / increases_ activity_ of / increases_ response_ to / decreases_ response_ to / related_ to – PPARA – actively_ involved_ in – cellular response to lipid – actively_ involves – CCL2 – genetically_ associated_ with – hepatic fibrosis* (Figure 1C). The answer subgraph contained 22 total edges, with 16 edges contributed by curated or text-mined knowledge sources and six co-occurrence edges contributed by OmniCorp. Notably, some, but not all, of the returned edges or predicates conveyed directionality in their relationships between nodes. For instance, while *biolink:related_to* is symmetric, *biolink:increases_response_to* is directional.

Numerous edges supported the first-hop relationship between clofibrate and PPARA; those edges served as validation for the ROBOKOP results, given that clofibrate is a known PPARA activator (Decara et al., 2020; National Center for Biotechnology Information and National Library of Medicine, 2024i). For instance, the Comparative Toxicogenomics Database (CTD) (Davis et al., 2023) contributed an edge that asserted “clofibrate increases the expression of *PPARA*,” with 12 supporting publications. One of the 12 publications was titled “Clofibrate, a peroxisome-proliferator, enhances reverse cholesterol transport through cytochrome P450 activation and oxysterol generation” (Guan et al., 2003). Additional curated edges supporting the relationship between clofibrate and PPARA were contributed by the knowledge sources DrugCentral (Avram et al., 2023), Drug Mechanism Database (Gonzalez-Cavazos et al., 2023), Human Metabolome Database (Wishart, 2024), and Binding Database (Skaggs School of Pharmacy and Pharmaceutical Sciences, 2025). Text Mining Provider (Baumgartner, 2024) contributed two edges supporting the relationship between clofibrate and PPARA, one with three supporting publications and the other with two supporting publications. One of the sentences from which the text-mined assertion was derived stated that “PPAR and its marker genes *Cyp4a10* and *Cyp4a14* were induced 2–4 fold by icariin and 4–8 fold by clofibrate” (Lu et al., 2014). OmniCorp contributed an “occurs together in literature with” edge in support of the relationship between clofibrate and PPARA, with four PubMed co-occurrence counts. The second hop included an edge contributed by Hetionet (HetioNet Team, 2025), which asserted that “PPARA is actively involved in the cellular response to lipid.” The third hop included an edge that was also contributed by Hetionet, which asserted that “the cellular response to lipid actively involves CCL2.” The fourth hop included an edge, which asserted that “CCL2 is genetically associated with hepatic fibrosis.” This edge was contributed by the primary knowledge source DisGeNET (Piñero et al., 2019) via the aggregator knowledge source Pharos (Kelleher et al., 2023). Finally, one of the edges contributed by OmniCorp connected *PPARA* and hepatic fibrosis and was supported by eight PubMed co-occurrences.

In addition to exploring the answer subgraph shown in Figure 1, we modified our queries to explore additional intermediary genes or proteins and biological processes or activities that matched the general structure of the query shown in Figure 1B. For instance, a query structured as *clofibrate – related_to – PPARA – related_ to – cellular response to lipid – related_ to – Gene or Protein – related_ to – hepatic fibrosis* returned 11 additional genes/proteins in addition to CCL2 (16 total answer subgraphs) (Table 1). Note that the query could be abbreviated to *PPARA – related_to – cellular response to lipid – related_ to – Gene Or Protein – related_ to – hepatic fibrosis* or even *cellular response to lipid – related_to – Gene Or Protein – related_ to – hepatic fibrosis*. These shorter queries will increase ROBOKOP’s response time. However, they will also change the scoring and ranking of answer subgraphs and the supporting evidence, such as the OmniCorp literature co-occurrence edges. Moreover, while theoretically any PPARA activator could be substituted for clofibrate in the first hop, that edge must exist within the graph in the first place. Given these considerations, we opted to maintain the full query structure for a direct comparison with the results shown in Figure 1.

**TABLE 1:** Genes/proteins identified by ROBOKOP in response to a query structured as *clofibrate – related_to – PPARA – related_ to – cellular response to lipid – related_ to – GeneOr Protein – related_ to – hepatic fibrosis*.

Gene abbreviation	Gene name	NCBI identifier
CCL2	C–C motif chemokine ligand 2 [*Homo sapiens*]	NCBIGene:6347 (National Center for Biotechnology Information and National Library of Medicine, 2024c)
IL-6	Interleukin 6 [*Homo sapiens*]	NCBIGene:3569 (National Center for Biotechnology Information and National Library of Medicine, 2024h)
TGFβ1	Transforming growth factor beta 1 [*Homo sapiens*]	NCBIGene:7040 (National Center for Biotechnology Information and National Library of Medicine, 2024k)
COL1A1	Collagen type I alpha 1 chain [*Homo sapiens*]	NCBIGene:1277 (National Center for Biotechnology Information and National Library of Medicine, 2024e)
CD14	Complement of differentiation 1 [*Homo sapiens*]	NCBIGene:929 (National Center for Biotechnology Information and National Library of Medicine, 2024d)
HDAC2	Histone deacetylase 2 [*Homo sapiens*]	NCBIGene:3066 (National Center for Biotechnology Information and National Library of Medicine, 2024f)
NCP1	NPC intracellular cholesterol transporter 1 [*Homo sapiens*]	NCBIGene:4864 (National Center for Biotechnology Information and National Library of Medicine, 2024m)
TNFRSF1B	TNF receptor superfamily member 1B [*Homo sapiens*]	NCBIGene:7133 (National Center for Biotechnology Information and National Library of Medicine, 2024l)
ABCB4	ATP-binding cassette subfamily B member 4 [*Homo sapiens*]	NCBIGene:5244 (National Center for Biotechnology Information and National Library of Medicine, 2024b)
PLAU	Plasminogen activator, urokinase [*Homo sapiens*]	NCBIGene:5328 (National Center for Biotechnology Information and National Library of Medicine, 2024m)
IL-12A	Interleukin 12A [*Homo sapiens*]	NCBIGene:3592 (National Center for Biotechnology Information and National Library of Medicine, 2024g)
STAT1	Signal transducer and activator of transcription 1 [*Homo sapiens*]	NCBIGene:6772 (National Center for Biotechnology Information and National Library of Medicine, 2024j)

Abbreviations: NCBI, National Center for Biotechnology Information.

Similarly, when we replaced “cellular response to lipid” in a query structured as clofibrate–related_to–PPARA–related_ to–Biological Process Or Activity–related_ to–CCL2–related_ to–hepatic fibrosis, ROBOKOP returned 30 additional biological processes or activities in addition to “cellular response to lipid” (31 total answer subgraphs) (Table 2). Examples include the following: “response to hypoxia,” “regulation of inflammatory response,” “regulation of leukocyte cell–cell adhesion,” “response to nutrient levels,” “regulation of response to wounding,” and “cellular response to hormone stimulus.”

**TABLE 2:** Biological processes or activities identified by ROBOKOP in response to a query structured as *clofibrate – related_to – PPARA – related_ to – Biological Process Or Activity - related_ to – CCL2 – related_ to – hepatic fibrosis*.

Biological process or activity	GO identifier
Protein binding	GO:0005515 (EMBL-EBI, 2024h)
Response to hypoxia	GO:0001666 (EMBL-EBI, 2024q)
Response to lipid	GO:0033993 (EMBL-EBI, 2024s)
Response to insulin	GO:0032868 (EMBL-EBI, 2024r)
Response to wounding	GO:0009611 (EMBL-EBI, 2024z)
Regulation of inflammatory response	GO:0050727 (EMBL-EBI, 2024k)
Regulation of cell adhesion	GO:0030155 (EMBL-EBI, 2024i)
Response to ethanol	GO:0045471 (EMBL-EBI, 2024p)
Response to steroid hormone	GO:0048545 (EMBL-EBI, 2024y)
Circulatory system development	GO:0072359 (EMBL-EBI, 2024f)
Regulation of leukocyte cell–cell adhesion	GO:1903037 (EMBL-EBI, 2024l)
Cellular response to lipid	GO:0071396 (EMBL-EBI, 2024c)
Cellular response to steroid hormone stimulus	GO:0071383 (EMBL-EBI, 2024e)
Response to peptide	GO:1901652 (EMBL-EBI, 2024x)
Cellular response to organic cyclic compound	GO:0071407 (EMBL-EBI, 2024d)
Response to organic cyclic compound	GO:0014070 (EMBL-EBI, 2024u)
Response to nutrient levels	GO:0031667 (EMBL-EBI, 2024t)
Regulation of defense response	GO:0031347 (EMBL-EBI, 2024j)
Anatomical structure formation involved in morphogenesis	GO:0048646 (EMBL-EBI, 2024a)
Cellular response to hormone stimulus	GO:0032870 (EMBL-EBI, 2024b)
Negative regulation of developmental process	GO:0051093 (EMBL-EBI, 2024g)
Response to oxygen levels	GO:0070482 (EMBL-EBI, 2024w)
Response to organonitrogen compound	GO:0010243 (EMBL-EBI, 2024v)
Regulation of response to wounding	GO:1903034 (EMBL-EBI, 2024m)
Regulation of response to external stimulus	GO:0032101 (EMBL-EBI, 2024n)
Response to decreased oxygen levels	GO:0036293 (EMBL-EBI, 2024o)
Response to peptide hormone	GO:0043434 (EMBL-EBI, 2025c)
Regulation of response to external stimulus	GO:0032102 (EMBL-EBI, 2024n)
Regulation of cell adhesion	GO:0022407 (EMBL-EBI, 2024i)
Animal organ morphogenesis	GO:0009887 (EMBL-EBI, 2025a)
Response to nutrient	GO:0007584 (EMBL-EBI, 2025b)

Abbreviations: GO, Gene Ontology.

## 4 Discussion

In this study, we described a real-world application case, in which we used ROBOKOP to substantiate the hypothesized relationship between the PPARA activator clofibrate and hepatic fibrosis. We framed our queries in the context of a pathogenic model proposed by Kim and Lee (2018) to explain the relationship between environmental exposures and the development of hepatic fibrosis. One answer subgraph returned by ROBOKOP suggested the following: *clofibrate – affects / increases_ expression_ of / increases_ activity_ of / increases_ response_ to / decreases_ response_ to / is_ related_ to – PPARA – is_ actively_ involved_ in – cellular response to lipid – actively_ involves – CCL2 – is_ genetically_ associated_ with – hepatic fibrosis*. Notably, the first-hop edge between clofibrate and PPARA was important to be established as an initial validation step, given that clofibrate is a known PPARA activator (Decara et al., 2020; National Center for Biotechnology Information and National Library of Medicine, 2024i). The full answer subgraph can be interpreted as follows: the cellular response to lipids actively involves CCL2, which is genetically associated with hepatic fibrosis; because PPARA is also actively involved with the cellular response to lipids, we can then infer that PPARA, upon activation by the herbicide clofibrate, is involved in hepatic fibrosis. Note that these inferences do not necessarily imply causality but rather suggest relationships to explain a hypothesized association between the PPARA activator clofibrate and hepatic fibrosis.

Kim and Lee (2018) described PPARA-activating therapeutics as a strategy to reduce hepatic steatosis in patients with non-alcoholic steatohepatitis via enhancement of β-oxidation. Others describe the role of PPARA agonists, such as fenofibrate and clofibrate, as lipid-lowering drugs in clinical practice (Qiu et al., 2023). Clofibrate, a relatively weak PPARA agonist (Krey et al., 1997), was approved for use as a lipid-lowering drug in 1963, but it was discontinued in 2002 due to concerns related to SIADH, a syndrome of excess secretion of antidiuretic hormone or vasopressin (Wikimedia Foundation Inc, 2024). Clofibrate and other fibrates have been used to treat patients with NAFLD, but their clinical efficacy has been limited (Choudhary et al., 2019). Chronic PPARA activation in rats has been associated with liver pathology (peroxisome proliferation) that ultimately leads to hepatocellular carcinoma, suggesting safety concerns for long-term environmental exposure to compounds such as clofibrate (Daynes and Jones, 2002). Clinically apparent liver injury may occur in a small proportion of patients receiving therapeutic doses, typically after 2–3 months of treatment, but the injury is generally mild and normally resolves with the discontinuation of the drug (Guan et al., 2003). Additionally, the potency of PPARA agonists is correlated with their ability to induce hepatocarcinogenesis after chronic treatment in rodent models (Peters et al., 2012). Thus, the literature contains evidence that PPARA may both contribute to and prevent liver disease, depending on factors such as dose, potency, duration of exposure, and species. ROBOKOP can help clarify these and other complex relationships by providing rich supporting evidence and provenance in query results, as well as enabling users to deeply explore answer subgraphs and iteratively refine queries—for example, by specifying increasingly specific predicates (e.g., *biolink:causes* instead of *biolink:related_to* when such an edge is present in the graph). However, ROBOKOP currently does not take into account quantitative factors such as dose, potency, or duration of exposure in queries, although it supports qualified queries and will return qualified edge attributes in results when available. Moreover, sequential time series of events are not supported by ROBOKOP. Species-specific results can be targeted by specifying certain node categories (e.g., *biolink:Gene*), but a key strength of ROBOKOP is its ability to incorporate cross-species knowledge when deriving results.

Although ROBOKOP results lack the granularity of the model put forward by Kim and Lee, the results established a relationship between clofibrate, PPARA, and hepatic fibrosis and captured several key features of the model. For instance, both the biological process “cellular response to lipid” and the immune mediator CCL2 (C–C motif chemokine ligand 2) (National Center for Biotechnology Information, National Library of Medicine, 2024a) are key events in the model put forward by Kim and Lee. CCL2 serves as a chemoattractant that targets hepatic stellate cells and recruits macrophages and monocytes to the site of liver injury, thereby contributing to hepatic fibrosis (Poulsen et al., 2022). Moreover, our additional queries identified genes/proteins of relevance to the Kim and Lee model, including TGFβ, TNFα, TNFRSF1B, L-6, and IL-12A. We also identified additional biological processes or activities of relevance to the Kim and Lee model, including “response to hypoxia,” “regulation of inflammatory response,” “regulation of leukocyte cell–cell adhesion,” “response to nutrient levels,” “regulation of response to wounding,” and “cellular response to hormone stimulus.” Many, but not all, of the genes/proteins and biological processes/activities tend to be associated with inflammation. This is not surprising, given that PPARA regulates various aspects of immune function, including the expression of inflammatory cytokines such as CCL2 (Gong et al., 2023; Devchand et al., 1996; Poynter and Daynes, 1998).

Notably, we were unable to link biological processes/activities and genes/proteins to cell types such as Kupffer cells or hepatic stellate cells, both of which were highlighted in the model proposed by Kim and Lee. This was because certain Biolink Model classes such as *biolink:Cell* and *biolink:CellularComponent* are currently underrepresented in ROBOKOP. We are in the process of capturing and integrating knowledge sources, such as cell–cell interaction database (Noh et al., 2023), which include additional entity types and relationships. By increasing the diversity of entity types available in ROBOKOP and the relationships between nodes, we will be able to better support the generation and exploration of more detailed AOPs and the concept of “computable AOPs” (Edwards, 2017). We recently implemented (since the original submission of this paper) basic AOP templates in the “load example” dropdown menu within the ROBOKOP UI. Although the current examples are simplistic, examining genes, biological pathways/processes, and phenotypic features relating a chemical exposure to a disease, they provide a generic template that users can build upon to construct their own AOPs in a computable form.

One strength of ROBOKOP is its ability to provide more specific discoveries compared to many other KG-based systems in general and large language models (LLMs) in particular (Thapa and Adhikari, 2023). We have been exploring the application of LLMs to generate multilevel toxicological narratives that track from the molecular initiating event to disease and cover a variety of levels of biological organization. The various LLMs that we have been using all tend to converge on a few well-studied genes/proteins, biological processes/activities, and highly utilized supporting knowledge sources, thus resulting in homogenization across disease processes and lacking specificity. ROBOKOP, in contrast, does not converge on only those well-studied genes/proteins and biological processes/activities but rather offers many suggested biological mechanisms and diverse supporting knowledge sources to explain a connection between chemical exposures and an adverse outcome, as demonstrated in this study.

An additional strength of ROBOKOP is the rich confidence, evidence, and provenance it provides to support answers, including statistical metrics, supporting publications (when available), and complete provenance trails. Few KG- or LLM-based systems offer full transparency in both established and inferred answers to user queries.

Another noteworthy feature of ROBOKOP is its user-driven approach to querying the ROBOKOP KG and exploring answer subgraphs. Navigating and interpreting KG is an art form—one that is entirely user-driven. This flexibility allows individual users to choose their queries and explore answer subgraphs based on their specific interests, thus allowing one user to focus on a particular insight that another user may not find interesting and *vice versa*. This powerful feature allows multiple users, each with their own scientific backgrounds and interests, to derive distinct biological insights from the same KG, thereby accelerating biomedical discovery.

In conclusion, the results of this study build upon our prior findings regarding the application of ROBOKOP in supporting biomedical discovery and hypothesis generation across diverse application areas, including environmental health and drug discovery (Bizon et al., 2019; Fecho et al., 2021; Korn et al., 2022; Morton et al., 2019), and they extend ROBOKOP‘s applicability to toxicology.

## Data Availability

Publicly available datasets were analyzed in this study. ROBOKOP and associated tools are openly available at https://robokop.renci.org/. The question-builder tool can be openly accessed at https://robokop.renci.org/question-builder. The ROBOKOP KG can be explored and downloaded at https://robokop.renci.org/api-docs/docs/automat/robokop-kg. A description of the knowledge sources that have been integrated in whole or in part in the ROBOKOP KG can be found at https://robokop.renci.org/api-docs/docs/category/automat. Finally, the GitHub repositories associated with ROBOKOP can be found at https://github.com/RobokopU24.
